# Derivation of a 3-Item Patient Health Questionnaire as a Shortened Survey to Capture Depressive Symptoms

**DOI:** 10.1001/jamanetworkopen.2025.22036

**Published:** 2025-07-21

**Authors:** Roy H. Perlis, Faith M. Gunning, Mauricio Santillana, Matthew A. Baum, James N. Druckman, Katherine Ognyanova, David Lazer

**Affiliations:** 1 Center for Quantitative Health, Massachusetts General Hospital, Boston; 2 Department of Psychiatry, Harvard Medical School, Boston, Massachusetts; 3 AI Editor, *JAMA Network Open*; 4 Department of Psychiatry, Weill Cornell Medicine, New York, New York; 5 Associate Editor, *JAMA Network Open*; 6 Network Science Institute, Northeastern University, Boston, Massachusetts; 7 Institute for Quantitative Social Science, Harvard University, Boston, Massachusetts; 8 John F. Kennedy School of Government, Harvard University, Cambridge, Massachusetts; 9 Department of Government, Harvard University, Cambridge, Massachusetts; 10 Department of Political Science, University of Rochester, Rochester, New York; 11 Department of Communication, School of Communication and Information, Rutgers University, New Brunswick, New Jersey

## Abstract

**Importance:**

Screening measures of depressive symptoms (eg, 9-item Patient Health Questionnaire [PHQ-9]) are increasingly used in surveys and remote applications, where shorter versions would be valuable.

**Objective:**

To derive shorter versions of the PHQ-9 that maximize the variability in total depressive symptom severity captured.

**Design, Setting, and Participants:**

This survey study used data from 4 waves of a 50-state nonprobability web-based survey conducted between November 2, 2023, and July 21, 2024. Survey respondents were aged 18 years or older. The first survey wave data were used to identify shortened question subsets capturing variance in the PHQ-9 and estimating a PHQ-9 score of 10 or higher. Resulting models (eg, 3-item version of the PHQ [PHQ-3]) were validated in subsequent survey waves.

**Main Outcome and Measure:**

Performance of PHQ-3 in the full sample and across subgroups of age, gender, race and ethnicity, and educational levels. Depressive symptom severity was measured with the PHQ-9 (total score range: 0-27, with a score ≥10 indicating moderate or greater depressive symptoms).

**Results:**

In the 4 survey waves, there were 96 234 total participants (mean [SD] age, 47.3 [17.1] years; 55 245 [57.4%] identifying as women). In the full sample, 4401 participants (4.6%) identified as Asian American, 12 699 (13.2%) as Black or African American, 9776 (10.2%) as Hispanic or Latino, and 65 309 (67.9%) as White individuals, with 4049 (4.2%) who identified as having other race or ethnicity. Among these participants, the mean (SD) PHQ-9 score was 6.5 (6.6), and 25 411 (26.4%) met the criteria for moderate or greater depressive symptoms (PHQ-9 score ≥10). The optimal 3-item version, PHQ-3, used items 2 (subject: depressed mood), 6 (self-esteem or failure), and 1 (interest), yielding a Cronbach α of 0.88 (95% CI, 0.88-0.88) and Pearson correlation with the PHQ-9 total score of 0.93 (95% CI, 0.93-0.94). At a threshold of 3 or greater, the PHQ-3 sensitivity was 0.98 (95% CI, 0.97-0.98) and specificity was 0.76 (95% CI, 0.75-0.76) for moderate or greater depressive symptoms. Performance was consistent across sociodemographic subgroups and survey waves.

**Conclusions and Relevance:**

This survey study of US adults identified a 3-item scale that remained highly correlated with the full PHQ-9 instrument. The reduced set of questions could enable more widespread and efficient incorporation of depressive symptom measurement in general population samples.

## Key Points

**Question:**

Can a shorter set of questions be used to estimate depressive symptom severity in the general population?

**Findings:**

In this survey study of 96 234 US adults, a reduced set of 3 items was derived from (and remained highly correlated with) the full 9-item Patient Health Questionnaire (PHQ). The shorter questionnaire showed utility in capturing depressive symptoms overall and across population subgroups.

**Meaning:**

The findings suggest that the 3-item PHQ could enable more widespread and efficient incorporation of depressive symptom measurement in general population samples.

## Teaser

This survey study assesses the performance of a shortened 3-item version of the 9-item Patient Health Questionnaire (PHQ-9) to estimate depressive symptom severity across patient age, gender, race and ethnicity, and educational levels.

## Introduction

The 9-item Patient Health Questionnaire (PHQ-9) was originally developed and validated as a screening measure for major depression. Subsequent studies suggest its utility for detecting depressive episodes across a range of contexts.^1^ For instance, the integration of patient-reported outcomes with electronic health records has been associated with the increased application of the PHQ in primary care settings. The PHQ also has been applied to measure depressive symptom severity in longitudinal population studies.^2,3^

However, while the PHQ-9 itself is brief, with increased deployment of patient-reported outcomes and growing desire to capture multiple symptom domains beyond depressive symptoms,^4,5^ self-reported scales in the aggregate can impose a substantial burden on patients. Faced with a long list of survey questions, some individuals may be tempted to speed through or to not respond at all. This temptation may be even greater outside of clinical settings, such as in population-based samples for epidemiologic studies, where attention is often fleeting.^6^ Lengthy question batteries also can adversely impact response rates^7^ and be financially costly in population-based samples,^8^ where survey costs can range from $750 to $1000 or more per question.^9^

To address the need for shorter assessment tools for integration with online surveys and telephone-based mobile applications, we drew on large-scale adult survey data that included the PHQ-9. We adopted the methods of prior studies that sought to shorten the PHQ-9 in aggregated screening studies using optimal test assessment (OTA), based on identifying a maximally informative subset of items.^10,11^ We further applied this large collection of data to examine whether these shorter subsets exhibited differences in performance across subgroups of age, gender, educational level, and race and ethnicity, an important gap in prior work. Finally, we tested the performance of gating on a single item, for applications wherein an even shorter measure is required. We aimed to derive shorter versions of the PHQ-9 that maximize the variability in total depressive symptom severity captured rather than trying to derive a clinical screening measure.

## Methods

### Study Design

We analyzed data from 4 waves of an internet survey conducted by a commercial aggregator of online panels for market research (PureSpectrum) between November 2, 2023, and July 21, 2024. All respondents provided electronic informed consent prior to answering survey questions and received remuneration for their participation. The Harvard University and Northeastern University Institutional Review Boards formally reviewed and approved this survey and the study design. We followed the American Association for Public Opinion Research (AAPOR) reporting guideline.^12^

The first survey wave (wave 29) occurred between November 2, 2023, and December 5, 2023, and the data were used to identify optimal questionnaire items and cutoffs. Subsequent survey waves (waves 30-32) occurred between December 21, 2023, and July 21, 2024, and their data were used to further characterize performance in the full sample and in subgroups defined by age, gender, educational level, and race and ethnicity. (Further details and discussion of survey methods are provided in Perlis et al^13,14^). Given that participants could return for more than 1 survey wave, we chose only the initial survey visit for any given respondent. Prior work with these surveys indicated that randomly selecting a survey wave or including multiple observations per individual yielded similar results.^14^ To ensure reliable human-generated responses, the survey included multiple attention checks as well as open-ended questions.

### Measures

We collected age, gender, educational level, and race and ethnicity via self-report. Race and ethnicity were selected from these categories: American Indian or Alaska Native, Asian American, Black or African American, Hispanic or Latino, Native Hawaiian or Other Pacific Islander, White, or other, with the opportunity to provide a free-text self-description. These data were initially collected to enable estimates of representativeness by US state^15^ and were applied here for subgroup analyses. As in prior work,^14^ we combined American Indian or Alaska Native, Native Hawaiian or Other Pacific Islander, and other race or ethnicity into a single category for analysis to facilitate the inclusion of smaller groups.

Depressive symptom severity was measured with the PHQ-9,^1,16^ including the diagnostic criteria for major depressive disorders in the *Diagnostic and Statistical Manual of Mental Disorders* (Fifth Edition), scored on a 0 (not at all) to 3 (nearly every day) Likert scale that yields a total score ranging from 0 to 27.^16^ Moderate or greater depressive severity was defined as a score of 10 or greater on the PHQ-9 based on prior reports.^1,16^

### Short-Form Derivation

To maximize comparability, we adapted our methods from a prior effort to generate a shortened 4-item version of the PHQ-9 in a clinical trials context.^10^ We began by applying a generalized partial credit model, a form of item response theory,^17^ implemented in the ltm package in R (R Project for Statistical Computing).^18^ This model examines item-level scores and relates them to a latent measure, representing total depressive symptoms. For each item, this model estimates a discrimination parameter and threshold parameters. These parameters allow a calculation of the test information function (TIF), which captures the degree of precision in measuring the latent trait.

We then sought to identify the items on the PHQ-9 that maximized the TIF for a given number of items. That is, the goal of this approach is to maximize the extent to which a smaller number of items capture the depressive symptom severity of the full 9-item version. Again, for consistency with prior work,^10^ we applied OTA to identify these items for a given survey length from 1 to 8^19^ implemented using the lpSolveAPI package in R.^20^

### Statistical Analysis

We aimed, primarily, to develop the shortest possible measure that retained reliability and correlation with the PHQ-9 and, secondarily, to examine sensitivity and specificity for the full PHQ-9–defined moderate depressive symptom threshold. Reliability and correlation were estimated using the Cronbach α and Pearson correlation coefficient (*r*)^21,22^ with the PHQ-9 summed score, respectively. We applied a bivariate random-effects model fit using the lme4 package^23^ to estimate sensitivity and specificity for moderate depressive symptoms via the PHQ-9 at each possible threshold. Optimal cutoff threshold was determined via the Youden J statistic.^24^

As we sought a shorter measure than existing ones,^10^ we examined the performance of a 3-item version of the PHQ (Quick PHQ-3 or QP-3; hereafter, PHQ-3) in the sample as a whole and in subgroups defined by sociodemographic features. We compared the performance of the PHQ-3 in each subgroup with the previously reported 4-item PHQ-Depression-4,^10^ testing that they were noninferior with a clinical tolerance of 0.02, and performance of the PHQ-Depression-4 was re-estimated in this dataset. The test statistic was estimated via bootstrapping^25^ (resampling with replacement) with 2000 bootstrap iterations, maintaining the proportion of respondents with and without moderate or greater depressive symptoms. Finally, we examined predictive validity for the PHQ-3 and the gated version by examining the area under the receiver operating characteristic curve (AUROC), estimating moderate or greater depressive symptoms at the second wave among respondents to the first wave of the survey, and compared that with the predictive validity of the full PHQ-9. For comparison, we also examined the performance of the 2-item PHQ (PHQ-2) used alone and as a screen in which the full PHQ-9 was completed only if the PHQ-2 score exceeded a threshold.

All analyses used R, version 4.3.2 (R Project for Statistical Computing).^26^ Two-tailed *P* = .05 indicated statistical significance.

## Results

Across the 4 survey waves, there were 96 234 total participants, with a mean (SD) age of 47.3 (17.1) years and including 55 245 (57.4%) identifying as women, 40 137 (41.7%) identifying as men, and 852 (0.9%) identifying as nonbinary (Table 1). In the full sample, 4401 participants (4.6%) identified as Asian American, 12 699 (13.2%) as Black or African American, 9776 (10.2%) as Hispanic or Latino, and 65 309 (67.9%) as White individuals, with 4049 individuals (4.2%) who identified as other race or ethnicity. The mean (SD) PHQ-9 score was 6.5 (6.6). A total of 25 411 participants (26.4%) met the criteria for at least moderate depressive symptoms (PHQ-9 score ≥10).

**Table 1.** Characteristics of Full Survey Cohort

Characteristic	No. (%) [N = 96 234]
Gender	
Woman	55 245 (57.4)
Man	40 137 (41.7)
Nonbinary	852 (0.9)
Age, mean (SD), y	47.3 (17.1)
Race and ethnicity^a^	
Asian American	4401 (4.6)
Black or African American	12 699 (13.2)
Hispanic or Latino	9776 (10.2)
White	65 309 (67.9)
Other^b^	4049 (4.2)
Educational level	
≤Some high school	3550 (3.7)
High school diploma	23 614 (24.5)
Some college	24 940 (25.9)
Bachelor’s degree	32 812 (34.1)
≥Master’s degree	11 318 (11.8)
Total PHQ-9 score, mean (SD)^c^	6.5 (6.6)
With moderate or greater depressive symptom severity	25 411 (26.4)
Survey wave	
Wave 29: November 2, 2023-December 5, 2023	27 933 (29.0)
Waves 30-32: December 21, 2023-July 21, 2024	68 301 (71.0)

Abbreviation: PHQ-9, 9-item Patient Health Questionnaire.

^a^ Race and ethnicity were self-reported by participants.

^b^ Other race and ethnicity include American Indian or Alaska Native, Native Hawaiian or Other Pacific Islander, and Other.

^c^ PHQ-9 score range: 0 to 27, with 10 or higher indicating moderate or greater depressive symptom severity.

We first estimated the TIF value for each PHQ-9 item (Table 2), followed by item selection using OTA for each survey length from 1 to 8. Test correlations and discrimination at the optimal threshold are summarized in Table 3. The optimal version, PHQ-3, used items 2 (subject: depressed mood), 6 (self-esteem or failure), and 1 (interest), yielding a Cronbach α of 0.88 (95% CI, 0.88-0.88) and Pearson *r* with the PHQ-9 total score of 0.93 (95% CI, 0.93-0.94). At a threshold of 3 or greater, the PHQ-3 sensitivity was 0.98 (95% CI, 0.97-0.98) and specificity was 0.76 (95% CI, 0.75-0.76).

**Table 2.** Patient Health Questionnaire–9 Items by Test Information Function^a^

PHQ-9 item	Subject	TIF	Full text
2	Depressed mood	1.831	Feeling down, depressed, or hopeless
6	Self-esteem or failure	1.423	Feeling bad about yourself, or [feeling] that you are a failure or have let yourself or your family down
1	Interest	1.289	Little interest or pleasure in doing things
7	Concentration	1.140	Trouble concentrating on things, such as reading the newspaper or watching television
4	Fatigue	1.081	Feeling tired or having little energy
5	Appetite	0.983	Poor appetite or overeating
9	Death or suicide	0.935	Thoughts that you would be better off dead, or [thoughts] of hurting yourself
8	Motor	0.834	Moving or speaking so slowly that other people could have noticed. Or the opposite, being so fidgety or restless that you have been moving around a lot more than usual
3	Sleep	0.765	Trouble falling or staying asleep, or sleeping too much

Abbreviations: PHQ-9, 9-item Patient Health Questionnaire; TIF, test information function.

^a^ Test items are preceded by “Over the last 2 weeks, how often have you been bothered by any of the following problems….”

**Table 3.** Performance of Shortened Patient Health Questionnaire Versions Selected via Optimal Test Assembly

Survey length	Items	Optimal TIF	(95% CI)		Optimal cutoff	(95% CI)			
			Cronbach α	Correlation, Pearson *r*		Sensitivity	Specificity	NPV	PPV
PHQ-1	2	1.831	NA	0.86 (0.86-0.86)	0	1.00 (1.00-1.00)	0.00 (0.00-0.00)	NA	0.26 (0.25-0.26)
PHQ-2	2, 6	3.254	0.86 (0.85-0.86)	0.91 (0.91-0.91)	2	0.95 (0.95-0.96)	0.74 (0.74-0.75)	0.98 (0.98-0.98)	0.56 (0.55-0.57)
PHQ-3	1, 2, 6	4.542	0.88 (0.88-0.88)	0.93 (0.93-0.94)	3	0.98 (0.97-0.98)	0.76 (0.75-0.76)	0.99 (0.99-0.99)	0.58 (0.57-0.59)
PHQ-4	1, 2, 6, 7	5.682	0.89 (0.89-0.90)	0.96 (0.96-0.96)	5	0.94 (0.93-0.94)	0.89 (0.89-0.90)	0.98 (0.97-0.98)	0.75 (0.74-0.76)
PHQ-5	1, 2, 4, 6, 7	6.763	0.90 (0.90-0.91)	0.98 (0.97-0.98)	6	0.96 (0.95-0.96)	0.91 (0.90-0.91)	0.99 (0.98-0.99)	0.78 (0.77-0.79)
PHQ-6	1, 2, 4, 5, 6, 7	7.746	0.91 (0.91-0.92)	0.98 (0.98-0.98)	7	0.98 (0.98-0.98)	0.92 (0.91-0.92)	0.99 (0.99-0.99)	0.80 (0.79-0.81)
PHQ-7	1, 2, 4, 5, 6, 7, 9	8.681	0.92 (0.91-0.92)	0.99 (0.99-0.99)	8	0.99 (0.99-0.99)	0.93 (0.93-0.93)	1.00 (1.00-1.00)	0.83 (0.82-0.84)
PHQ-8	1, 2, 4, 5, 6, 7, 8, 9	9.515	0.92 (0.92-0.92)	0.99 (0.99-0.99)	9	0.99 (0.99-0.99)	0.97 (0.96-0.97)	1.00 (1.00-1.00)	0.91 (0.90-0.91)
PHQ-1 of PHQ-3	if 2 > 0 then 6, 1	2.334^a^	NA	0.91 (0.91-0.91)	3	0.94 (0.93-0.94)	0.81 (0.81-0.82)	0.97 (0.97-0.98)	0.64 (0.63-0.65)
PHQ-2	PHQ-2 screen 1, 2	3.119	0.86 (0.85-0.86)	0.90 (0.89-0.90)	2	0.95 (0.95-0.96)	0.74 (0.74-0.75)	0.98 (0.98-0.98)	0.56 (0.55-0.57)
PHQ-2 of PHQ-9	PHQ-2 + 7 if 1 + 2 > 2 then all^b^	4.722^a^	NA	0.89 (0.89-0.90)	10	0.73 (0.72-0.74)	1.00 (1.00-1.00)	0.92 (0.91-0.92)	1.00 (1.00-1.00)

Abbreviations: NA, not applicable; NPV, negative predictive value; PHQ, Patient Health Questionnaire; PPV, positive predictive value; TIF, test information function.

^a^ TIF was calculated as weighted mean of smaller and larger scale (eg, 1-item and 3-item, or 2-item and 7-item).

^b^ 6251 of 27 933 = 22.4%.

We also examined the performance of the PHQ-3 in which the most informative item (2) was used to gate completion of the next 2 items (6 and 1); that is, a nonzero score on item 2 prompted the collection of 2 more items. The gated item version yielded modestly diminished correlation (Pearson *r* = 0.91; 95% CI, 0.91-0.91), with a sensitivity of 0.94 (95% CI, 0.93-0.94) and specificity of 0.81 (95% CI, 0.81-0.82). For this version, 34 563 of 67 179 participants (51.4%) in subsequent survey waves would have completed only 1 item; the mean (SD) number of items was 1.97 (1.00).

For comparison, we also examined the performance of the PHQ-2 alone, yielding a Pearson *r* of 0.90 (95% CI, 0.89-0.90). When used as a screen, in which the full PHQ-9 was completed only if the 2-item score exceeded a threshold, the PHQ-2 yielded a Pearson *r* of 0.90 (95% CI, 0.89-0.90).

We used the 3 subsequent survey waves to again examine the performance of the PHQ-3 in the sample as a whole and in individual subgroups defined by sociodemographic features (Figure; eTable in Supplement 1). Sensitivity exceeded 0.94 for all subgroups except individuals aged 65 years or older (0.93; 95% CI, 0.92-0.95); minimum specificity was 0.71 (95% CI, 0.68-0.73) for individuals with the lowest educational level (≤some high school). For the sample as a whole and all subgroups, the PHQ-3 was statistically noninferior to the previously reported sensitivity and specificity of the PHQ-Depression-4 (eTable in Supplement 1).

**Figure.**
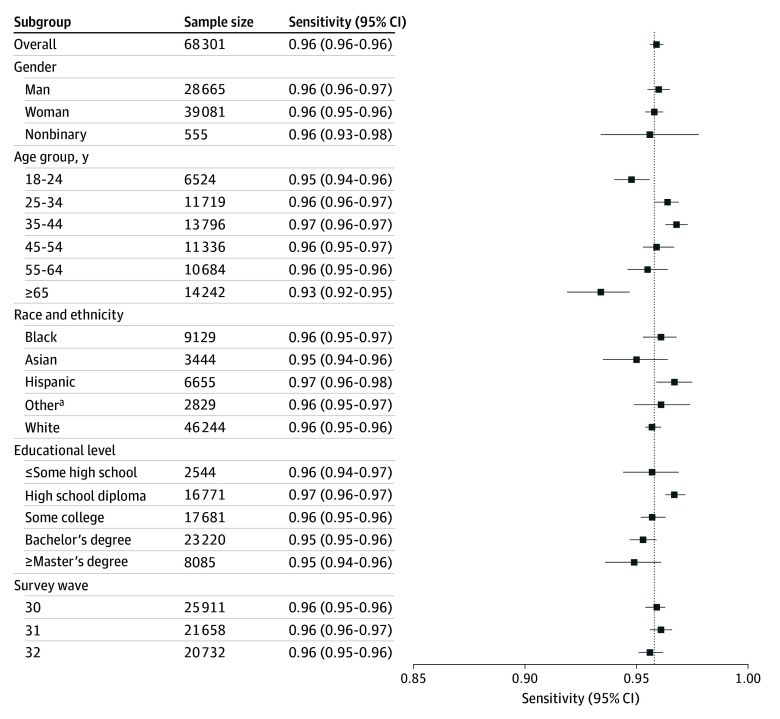
Sensitivity Estimates of the 3-Item Patient Health Questionnaire by Subgroup Error bars represent 95% CIs. ^a^Other self-reported race and ethnicity include American Indian or Alaska Native, Native Hawaiian or Other Pacific Islander, and other.

To examine predictive validity for subsequent moderate or greater depressive symptoms, we compared the AUROC using the shortened item score at wave 29 to estimate the presence of moderate or greater depressive symptoms at wave 30. The AUROC was 0.83 (95% CI, 0.81-0.84) for the PHQ-3, 0.80 (95% CI, 0.79-0.82) for the gated version, and 0.84 (95% CI, 0.82-0.86) for the PHQ-9, indicating modest but significant differences (*P* < .001).

## Discussion

Applying survey data from more than 96 000 US individuals aged 18 years or older, we derived shorter versions of the PHQ-9 that achieved promising correlations with the full measure. In particular, we identified the 3-item PHQ that exhibited similar performance to that of a previously reported PHQ-Depression-4.^10^ A single-item gated derivative was modestly diminished and had the advantage that 51.4% of respondents completed only a single item. While our primary goal was to capture depressive symptom severity and not to screen for moderate depressive symptoms, the PHQ-3 yielded sensitivity for the PHQ-9–defined moderate depressive symptoms that exceeded 90% in all subgroups, suggesting its applicability for diverse populations. This feature is particularly relevant given that prior work has suggested the need to consider alternate PHQ-9 thresholds in multiracial and multiethnic populations.^27^ Performance was stable across multiple survey waves, and the shorter measures estimated subsequent-visit presence of moderate or greater depressive symptoms. (For clinical contexts in which maximizing PPV is required, the results underscore the continued utility of including as many items as possible.)

By design, we followed the approach described by Ishihara et al,^10^ which applies robust standards to derive shortened measures using OTA. While results of the present study are generally similar to those previously reported, individual item scores differ somewhat in TIF. Ishihara et al^10^ found item 9, which captures thoughts of death or suicide, to be the most predictive, followed by 2, 1, and 6. In this study, the lower TIF for item 9 likely reflects that our cohort was not clinical but rather representative of the general US population. As such, levels of suicidality would likely be substantially lower than in clinical trial samples, for example, along with overall levels of severity. Thus, neither measure is more correct per se; rather, the optimal configuration of items likely depends on the application. Our intent was to derive a measure suited for rapid administration to capture symptom severity in general surveys, and not for specific clinical settings. While item 9 is often omitted from the PHQ,^10^ yielding the 8-item PHQ, screening for suicide risk remains a critical part of a clinical assessment.^28^

### Limitations

Our study has limitations. First, we did not have a gold standard clinician rating to derive true sensitivity and specificity. Instead, we compared the measures with the full PHQ-9 instrument, for which sensitivity and specificity has been repeatedly established^1^ despite recognized limitations, such as combining psychomotor agitation and retardation in a single item,^29^ and with the previously validated PHQ-4.^11^ Prior to considering its application in clinical settings, a critical next step is to examine the performance of the PHQ-3 subset in clinical samples, including clinical interview. Performance of other subsetted versions depends on the particular interview applied to derive a gold standard.^11^ However, 26.4% of the survey respondents reported moderate or greater depressive symptoms, reinforcing that large population-based samples may still be informative about phenomenology outside of specific clinical settings.

Second, the use of an internet survey–based sample introduces biases related to propensity to respond to online surveys. However, these data were drawn from a large national survey, which has been repeatedly shown to reflect more standard probability-based surveys.^30,31^ The survey incorporated attention checks and free-text answers to allow exclusion of automated responses and other low-attention strategies. Another strength of the internet design is that it allows sampling of some populations (older and racially and ethnically minoritized individuals, for example) who are often underrepresented in clinical samples.

## Conclusions

In this survey study of US adults, we derived a 3-item scale that remained highly correlated with the full PHQ-9 instrument and maintained this performance across subgroups of age, gender, race and ethnicity, and educational levels. While a shortened scale cannot capture the full range of the PHQ-9, it may facilitate more widespread and efficient investigation of psychiatric symptoms in general population samples when participant burden and/or data collection expense must be minimized.

## Notes

Supplement 1. **eTable.** QP-3 Performance in the Overall Sample and Subgroups, Overall and In Comparison to PHQ-4

Supplement 2.
Data Sharing Statement
