# Supplementary material for: Derivation of a 3-Item Patient Health Questionnaire as a Shortened Survey to Capture Depressive Symptoms
Source: JAMA Netw Open. 2025 Jul 21;8(7):e2522036. doi: 10.1001/jamanetworkopen.2025.22036 (PMC12281233; doi:10.1001/jamanetworkopen.2025.22036)
Supplement: Supplement 1. — eTable. QP-3 Performance in the Overall Sample and Subgroups, Overall and In Comparison to PHQ-4 [file jamanetwopen-e2522036-s001.pdf]

Supplemental Online Content

Perlis RH, Gunning FM, Santillana M, et al. Patient Health Questionnaire derivation as a shortened survey to capture depressive symptoms. *JAMA Netw Open*. 2025;8(7):e2522036. doi:10.1001/jamanetworkopen.2025.22036

eTable. QP-3 Performance in the Overall Sample and Subgroups, Overall and In Comparison to PHQ-4

This supplemental material has been provided by the authors to give readers additional information about their work.

© 2025 Perlis RH et al. *JAMA Network Open*

eTable. QP-3 Performance in the overall sample and subgroups, overall and in comparison to PHQ-4

**Subgroup**	**N**	**Sensitivity**	**Specificity**	**NPV**	**PPV**	**Sensitivity (non-inferiority p)(a)**	**Specificity (non-inferiority p)**
Overall	68301	0.959 (0.956-0.962)	0.807 (0.804-0.811)	0.982 (0.981-0.983)	0.644 (0.640-0.648)	1.00	1.00
Males	28665	0.960 (0.955-0.965)	0.822 (0.817-0.827)	0.985 (0.984-0.987)	0.619 (0.612-0.626)	0.99	1.00
Females	39081	0.958 (0.954-0.962)	0.797 (0.792-0.801)	0.979 (0.977-0.981)	0.656 (0.651-0.661)	1.00	1.00
Nonbinary	555	0.956 (0.934-0.978)	0.684 (0.626-0.740)	0.920 (0.883-0.957)	0.805 (0.777-0.835)	1.00	1.00
18-24	6524	0.948 (0.940-0.956)	0.723 (0.708-0.738)	0.943 (0.935-0.952)	0.741 (0.731-0.751)	0.92	1.00
25-34	11719	0.964 (0.958-0.969)	0.729 (0.719-0.739)	0.970 (0.965-0.974)	0.690 (0.682-0.698)	1.00	1.00
35-44	13796	0.968 (0.963-0.973)	0.753 (0.744-0.762)	0.980 (0.977-0.983)	0.654 (0.646-0.662)	1.00	1.00
45-54	11336	0.959 (0.953-0.967)	0.783 (0.774-0.792)	0.980 (0.977-0.984)	0.632 (0.622-0.641)	1.00	1.00
55-64	10684	0.955 (0.946-0.964)	0.839 (0.831-0.846)	0.989 (0.986-0.991)	0.559 (0.547-0.570)	1.00	1.00
65+	14242	0.934 (0.919-0.947)	0.907 (0.902-0.912)	0.993 (0.992-0.995)	0.480 (0.467-0.494)	1.00	1.00
Black	9129	0.961 (0.953-0.968)	0.803 (0.793-0.812)	0.982 (0.979-0.986)	0.643 (0.632-0.654)	1.00	1.00
Asian	3444	0.950 (0.935-0.964)	0.801 (0.786-0.817)	0.978 (0.972-0.984)	0.634 (0.615-0.651)	0.55	1.00
Hispanic	6655	0.967 (0.959-0.975)	0.784 (0.773-0.796)	0.980 (0.975-0.985)	0.684 (0.672-0.696)	1.00	1.00
Other	2829	0.961 (0.949-0.974)	0.784 (0.765-0.802)	0.978 (0.971-0.985)	0.668 (0.649-0.687)	0.97	1.00
White	46244	0.957 (0.954-0.961)	0.813 (0.809-0.817)	0.982 (0.981-0.984)	0.637 (0.632-0.642)	1.00	1.00
Some High School or Less	2544	0.957 (0.944-0.969)	0.708 (0.684-0.731)	0.956 (0.944-0.968)	0.713 (0.696-0.729)	0.98	1.00
High School Graduate	16771	0.967 (0.963-0.972)	0.777 (0.769-0.785)	0.979 (0.976-0.982)	0.687 (0.679-0.695)	1.00	1.00
Some College	17681	0.957 (0.952-0.963)	0.793 (0.786-0.800)	0.978 (0.975-0.981)	0.660 (0.653-0.668)	1.00	1.00
College Degree	23220	0.953 (0.947-0.959)	0.826 (0.821-0.831)	0.985 (0.983-0.987)	0.600 (0.592-0.607)	1.00	1.00
Graduate Degree	8085	0.949 (0.936-0.961)	0.855 (0.846-0.863)	0.989 (0.986-0.992)	0.550 (0.535-0.565)	0.93	1.00
Wave 30	25911	0.959 (0.954-0.963)	0.800 (0.794-0.806)	0.981 (0.979-0.983)	0.640 (0.634-0.647)	1.00	1.00
Wave 31	21658	0.961 (0.956-0.966)	0.811 (0.805-0.817)	0.982 (0.979-0.984)	0.663 (0.656-0.670)	1.00	1.00
Wave 32	20732	0.956 (0.951-0.962)	0.813 (0.807-0.819)	0.983 (0.980-0.985)	0.628 (0.620-0.636)	1.00	1.00

(a) P-values refer to comparisons with the PHQ-4.

© 2025 Perlis RH et al. *JAMA Network Open*
