# Supplementary material for: Derivation of a 3-Item Patient Health Questionnaire as a Shortened Survey to Capture Depressive Symptoms
Source: JAMA Netw Open. 2025 Jul 21;8(7):e2522036. doi: 10.1001/jamanetworkopen.2025.22036 (PMC12281233; doi:10.1001/jamanetworkopen.2025.22036)
Supplement: Supplement 2. — Data Sharing Statement [file jamanetwopen-e2522036-s002.pdf]

Data Sharing Statement

Perlis. Derivation of a 3-Item Patient Health Questionnaire as a Shortened Survey to Capture Depressive Symptoms. *JAMA Netw Open*. Published July 21, 2025. doi:10.1001/jamanetworkopen.2025.22036

Data
Data available: Yes
Data types: Deidentified participant data
How to access data: covidstates.org
When available: beginning date: 07-01-2025

Supporting Documents
Document types: None

Additional Information
Who can access the data: Researchers whose proposed use of the data has been approved
Types of analyses: For a specified purpose
Mechanisms of data availability: After approval of proposal with signed DUA. Deidentified survey data are available from the corresponding author for non-commercial use.
